# Clinical and Paraclinical Characteristics of Endobronchial Pulmonary Squamous Cell Carcinoma—A Brief Review

**DOI:** 10.3390/diagnostics13213318

**Published:** 2023-10-26

**Authors:** Radu Serban Matache, Camelia Stanciu-Gavan, Daniel Pantile, Adrian Mihail Iordache, Andreea Octavia Bejgăneanu, Crenguța Sorina Șerboiu, Alexandra Floriana Nemes

**Affiliations:** 1 Department of Thoracic Surgery, “Marius Nasta” Institute of Pneumophtiziology, 050159 Bucharest, Romania; radu.matache@gmail.com; 2 Department of Thoracic Surgery, “Doctor Carol Davila” Central Military Emergency University Hospital, 010825 Bucharest, Romania; 3 Laboratory Department, Fundeni Clinical Hospital, 022328 Bucharest, Romania; 4 Department of Cellular, Molecular Biology and Histology, Carol Davila University of Medicine and Pharmacy, 050474 Bucharest, Romania; 5 Department of Radiology and Medical Imaging, University Emergency Hospital, 050098 Bucharest, Romania; 6 Department of Neonatology, Louis Turcanu Clinical Emergency Hospital for Children, 300011 Timisoara, Romania

**Keywords:** endobronchial squamous cell carcinoma, autofluorescence bronchoscopy

## Abstract

Background: Endobronchial squamous cell carcinoma is one of the most common types of tumors located inside the tracheobronchial tree. Patients often present in advanced stages of the disease, which most often leads to a targeted therapeutic attitude of pneumonectomy. Practicing lung parenchyma-preserving surgery led us to undertake this review. Materials and methods: We used three search platforms—SCIENCE, MEDLINE, and PubMed—in order to identify studies presenting case reports, investigations, and reviews on endobronchial squamous cell carcinoma. We identified the clinical and paraclinical features of endobronchial squamous cell carcinoma. All the selected articles were in English and addressed the clinical criteria of endobronchial squamous cell carcinoma, autofluorescence bronchoscopy in endobronchial squamous cell carcinoma, imaging features of endobronchial squamous cell carcinoma, blood tumor markers specific to lung squamous cell carcinoma, and histopathological features of endobronchial squamous cell carcinoma. Results: In total, 73 articles were analyzed, from which 48 articles were selected as bibliographic references. We present the criteria used for the identification of endobronchial squamous cell carcinoma in order to highlight its main characteristics and the most reliable technologies that can be used for the detection of this type of cancer. Conclusions: The current literature review highlights the clinical and paraclinical characteristics of endobronchial squamous cell carcinoma. It aims to open new paths for research and early detection with respect to the frequent practice of lung parenchymal preservation surgery.

## 1. Introduction

While endobronchial squamous cell carcinoma is a relatively rare entity within bronchopulmonary cancer [1,2], it is one of the most common carcinomas located centrally, inside the tracheal and bronchial lumen (the main bronchus and lobar bronchi), thus causing their partial or complete obstruction [1,3]. One of the most frequent clinical manifestations is hemoptysis [4], so the condition is referred to as a bleeding neoplasm. Other known associated symptoms are cough, recurrent pneumonia, wheezing, and chest pain [3,4]. The condition is more frequent among smokers [5]. High levels of serological tumor markers frequently found in squamous cell carcinoma include SCC-Ag (squamous cell carcinoma-associated antigen), Cyfra 21-1 (cytokeratin 19 fragment 21-1 antigen), CEA (carcinoembryonic antigen), and TrxR (Thioredoxin reductase) [6,7,8]. Computed tomography shows no characteristic signs of squamous cell carcinoma. Common means used for identifying endobronchial tumors include the cutoff sign [5], intertumoral air bronchogram, and Tsuboi classification to specify the type of intraluminal obstruction [9]. Bronchoscopy highlights changes in the mucosa, with several degrees of evolution from low-grade dysplasia (mild and moderate) to high-grade (severe and in situ carcinoma) and invasive carcinoma [10]. Bronchoscopy also enables specification of the features of endobronchial tumors. These include whether they are nodular, whether they are polypoid lesions, whether they have a large implantation base, the nature of bleeding, their intrabronchial location, and the degree of stenosis [11]. Through bronchoscopy, biological samples of tumor tissue can be taken to specify the anatomopathological and immunohistochemical profile [3], and to enable testing for the determination of immune cells, including the determination of PDL-1 [12]. From an anatomopathological perspective, squamous cell carcinoma constitutes about 20% of bronchopulmonary tumors, usually manifested as a central lesion [13].

Microscopically, squamous cell carcinoma has several characteristics, including specific keratinization and intercellular bridges, having a solidly involved growth pattern with hyperchromatic nuclei, and with the tumor cells lacking a glandular structure or mucin production [13].

This retrospective study aims to identify the clinical and paraclinical signs of squamous cell carcinoma to enable the early diagnosis and initiation of personalized therapy with the goal of sparing lung parenchyma and increasing the life expectancy of patients.

## 2. Materials and Methods

We reviewed the Guidelines on Reviews and Meta-Analyses (PRISMA, Canadian Institutes of Health Research; Universita di Modena e Reggio Emilia, Italy; Cancer Research UK) to ensure accurate reporting [14]. We used the recommendations from the preferred reporting elements systematically for the selection process.

### 2.1. Search Strategy

We used three online databases, SCIENCE, MEDLINE, and PubMed, from 1990, to identify studies presenting case reports, studies, and reviews of endobronchial squamous cell carcinoma. We analyzed the clinical and paraclinical features of endobronchial squamous cell carcinoma. We used the following search terms in English: clinical criteria of endobronchial squamous cell carcinoma, autofluorescence bronchoscopy in endobronchial squamous cell carcinoma, imaging characteristics of endobronchial squamous cell carcinoma, blood tumor markers specific to lung squamous cell carcinoma, and anatomopathological characteristics of endobronchial squamous cell carcinoma. We identified several relevant specialized articles from which we extracted important information. The information obtained was synthesized according to inclusion and exclusion criteria, which are outlined below.

### 2.2. Selection Criteria

The authors independently analyzed the title, abstract, and full text of the selected articles for each type of feature. The articles’ eligibility for inclusion in this systematic review was assessed. The authors applied inclusion and exclusion criteria to determine if the studies identified were eligible. Only articles considered eligible were studied. Reading and information extraction were performed in accordance with the main aims and objectives of the review.

### 2.3. Inclusion and Exclusion Criteria

As we aimed to analyze the clinical and paraclinical characteristics of patients with squamous cell endobronchial lung carcinoma, only studies fulfilling the following criteria were included:

(a) Inclusion of patients with endobronchial squamous cell carcinoma only;
(b) Original reports of research studies describing tomographic images or laboratory and/or clinical findings for this category of patient;
(c) Studies published in a journal in English;
(d) The study included features of autofluorescence bronchoscopy;
(e) Studies published from 2005 to date;
(f) Studies reporting autofluorescence bronchoscopy, case reports, retrospective studies on pathologic anatomy, clinical reports, and CT scans;
(g) Studies that included laboratory analyses of tumor markers in squamous cell carcinoma.

We excluded the following studies:

(a) Studies that did not report original data or use clear diagnostic criteria;
(b) Editorials, comments, and opinions;
(c) Letters and conference abstracts;
(d) Scientific papers that described operative techniques for squamous bronchopulmonary cancer.

### 2.4. Data Extraction

The authors independently screened the titles and abstracts of the identified articles. Articles were divided into the following categories: clinical characteristics, tumor markers, characteristics of autofluorescence bronchoscopy, and imaging characteristics of CT scans. Articles were reviewed to determine inclusion criteria. The operative indication elements for lung parenchymal-sparing surgery and the opportunity for resection with broncho-anastomosis were analyzed.

## 3. Results

In total, 73 articles were analyzed, from which 48 articles were selected. These were obtained through SCIENCE, MEDLINE and PubMed electronic searches. They were distributed as follows: clinic—6 citations, case reports—11, autofluorescence bronchoscopy—13, computed tomography—15, biomarkers—14, pathological anatomy—4. Table 1 summarizes the most representative articles that we reviewed. Of the 48 cited articles, only 19 presented diagnostic yield, specificity, and sensitivity. In total, eight retrospective studies, four observational studies, three descriptive, two prospective, one meta-analysis, and one review were identified.

### 3.1. Study Characteristics

**Clinical findings**: The symptomatology described in the literature is specified for endobronchial tumors [2]. Clinical presentation of patients with endobronchial squamous cell carcinoma revealed cough, chest pain, shortness of breath, blood in sputum, wheezing, hoarseness, recurrent chest infections (including bronchitis and pneumonia), weight loss and loss of appetite, and fatigue [4], post-obstructive pneumonia, wheezing, and hemoptysis [26,27]. Omar Elsaca et al. specified that squamous cell carcinomas (SCCs) are the most common type of central tumor, and can cause cough, dyspnea, atelectasis, post-obstructive pneumonia, wheezing, and hemoptysis [26]. 

**Laboratory findings**: In patients with squamous cell carcinoma, hypercalcemia is frequently encountered [26,28]. The analysis of blood tumor markers revealed the presence of SCC-Ag, CYFRA21-1 [6,15,16,17], CEA, and TrxR [6,7]. Although uncharacteristic of this type of cancer, elevated levels of CA 125 have been reported [15]. The blood determinations of these tumor markers can be performed by ELISA biochemical tests, electrochemical determinations on different sensors. Studies on stochastic sensors are known. Stochastic sensors based on maltodextrins with different dextrose equivalent were proposed for the assay of three lung-cancer biomarkers: neuron-specific enolase, carcinoembryonic antigen, and epidermal growth factor receptor. The two sensors proposed can determine simultaneously NSE, CEA, and HER-1 in whole-blood samples (qualitative and quantitative), with recoveries higher than 97.00%. This screening test may serve for fast and early detection of lung cancer [29]. Biochemical analysis by ELISA technique has 50% sensitivity at >90% specificity [30].

A special place is represented by the determination of serological tumor markers for PD-L1 immunity, which demonstrated the superiority of PD-1/PD-L1 inhibitors for patients with advanced squamous NSCLC compared to chemotherapy [12,31,32]. Rui-Lian Chen et al. conducted this meta-analysis to investigate the efficacy of PD-1/PD-L1 inhibitors versus chemotherapy for squamous NSCLC patients. Their study included 11 clinical trials involving 3112 patients, which compared the efficacy of PD-1/PD-L1 inhibitors with chemotherapy for advanced squamous NSCLC patients. They observed that PD-1/PD-L1 inhibitors significantly improved OS and PFS of advanced squamous-cell lung cancer when compared with chemotherapy [31]. 

The analyzed articles for tumor markers were 6, which included both separate markers and groups of markers. In total, 12 markers were found. The analysis of these articles is represented in Table 2.

In the analyzed studies, 6 groups of tumor markers were found and analyzed together, depending on the result of which the diagnosis was established. 

### 3.2. Anatomopathological Characteristics

Anatomopathological characteristics: macroscopically squamous cell carcinomas are located centrally, frequently at the tracheo-bronchial level, along the major airways, exophytic [13], with a tendency to exfoliate [28], frequently on the membranous area [33], and they show signs of bleeding [3]. Microscopically, cellular changes extend to the entire epithelium of the airways but without reaching the surface. Lesions progressing to CIS show grossly aberrant cytology (including patchy chromatin, variable nuclear size and shape, dyskaryosis, and other abnormal nuclear shapes) that extend throughout the airway epithelium but do not infiltrate the basement membrane [34]. Images of sheets or lobules composed of polygonal malignant cells with eosinophils, cytoplasm, and pleomorphic nuclei, with atypical mitosis, along with the existence of pearls of keratosis and the presence of intercellular junctions, have also been encountered [18]. Omar Elsaca et al. stated that acinar, papillary or micropapillary, and leptic or solid growth patterns represent most of the neoplastic gland production in squamous carcinoma [26]. The presence of keratin synthesis by tumor cells, which may also include intercellular desmosomes, is used to diagnose squamous cell carcinoma [26,35]. The gold standard for confirmation of neoplasia by bronchoscopy remains biopsy and histopathological examination, with a positive diagnosis rate in 82% of cases [10].

### 3.3. Immunohistochemical Markers

Immunohistochemical markers specific to squamous cell carcinoma are p40, CK5/6, and TP63 (p63) [36]. These were analyzed by Omar Elsaca et al.: the expression of p40, p63, CK5, and desmoglein appeared in squamous cell carcinoma on immunohistochemical examination [26]. Squamous cell carcinomas confined to the bronchial wall are also known to exhibit two distinct patterns of growth: superficial spread and endobronchial mass lesions. In these cases, the immunohistochemical expression of p53 and Ki-67 is correlated with survival rates [37].

### 3.4. CT Findings

CT findings on a computed-tomography scan (CT) indicate that primary endobronchial malignancies manifest as a polypoid lesion, a focal sessile lesion, eccentric narrowing of the airway lumen, or circumferential wall thickening [38]. Lung squamous cell carcinoma has a higher incidence of central localization with internal cavities, compared to the rest of the lung cancers [39]. Occasionally, a CT examination of endobronchial squamous carcinomas highlights a localized bronchial thickening with a long outgrowth, plated on the wall of the bronchus for about 5 cm [19]. Central mass lesions may show either sheath or occlusion of the segmental or lobar bronchus/endobronchial component or sheath of adjacent vessels [40]. As a distinct sign of endobronchial tumors, we frequently encounter “the sign of the bronchus” [20]. Chang Min Park stated in the study, “Tumors in the Tracheobronchial Tree: CT and FDG PET Features”, that in computed tomography (CT), primary malignant tumors manifest as a polypoid lesion, a focal sessile lesion, with eccentric narrowing of the airway lumen, or circumferential wall thickening. Because SCC arises from the surface epithelium, the tumor surface is typically irregular. During fluorine 18 fluorodeoxyglucose (FDG) positron emission tomography (PET), most squamous cell carcinomas show high uptake, whereas adenoid cystic carcinoma and mucoepidermoid carcinoma show variable uptake levels, depending on the grade of differentiation [38]. Yoonah Song conducted a study on 310 patients who presented with lung squamous cell carcinoma simulating benign infectious or inflammatory diseases. Pulmonary squamous cell carcinoma may present as localized, long, continuous, bronchial thickening on CT [19]. 

Only four representative scientific reports were found and analyzed. Bronchial sign, bronchial cutoff sign, polypoid lesion, focal lesion, and sessile lesions were studied, with their sensitivity and specificity rates. All analyzed elements are specified in Table 3.

### 3.5. Bronchoscopy Findings

Bronchoscopy with autofluorescence and fluoroscopy detects precancerous bronchial lesions located at the level of the bronchial tree [10,41]. During these examinations, pathological tissue appears reddish-brown in color, while invasive lesions appear as defects easily recognized by bronchial fluorescence [42]. These characteristics help the thoracic surgeon make the best therapeutic decision of tumor resection while sparing lung parenchyma. Autofluorescence bronchoscopy allows the detection of pathological changes by using spectral differences in fluorescence and absorption properties at different levels of normal versus dysplastic epithelium [42]. 

### 3.6. Flexible Autofluorescence Bronchoscopy

Flexible autofluorescence bronchoscopy (AFB) is necessary for the diagnosis of endobronchial tumors. This examination highlights mucosal changes with several degrees of evolution—low-grade (mild and moderate) dysplasia, high-grade (severe and in situ carcinoma), and invasive carcinoma [9]. It also evaluates suspicious endobronchial tumors, malignant changes, by detecting irregularities in bronchial mucosa, nodular or polypoid lesions, and carina thickening [10]. Occasionally, there may also be false-positive imaging aspects of neoplasm—these are areas of inflammation—or a thickened epithelium [21]. Malignant tissue on autofluorescence examination has much less of a green color and may appear as shades of red, brown, or magenta, depending on the AFB system used [43]. Xiaoxuan Zheng et al. conducted a retrospective study on 218 cases with 1208 biopsies, using white-light bronchoscope and autofluorescence bronchoscopy, in “Application of Quantitative Autofluorescence Bronchoscopy Image Analysis Method in Identifying Bronchopulmonary Cancer”. The paper specified the characteristics of the technique: white-light bronchoscope associated with autofluorescence bronchoscopy was able to differentiate between a benign and malignant lesion with a high sensitivity, specificity, positive predictive value, and negative predictive value (92.1%, 59.3%, 87.3%, and 71.1%, respectively) [22].

### 3.7. Other Bronchoscopy Techniques 

Other bronchoscopy techniques performed for the detection of endobronchial squamous carcinoma are narrow-band imaging bronchoscopy (NBI), confocal laser endomicroscopy and laser Raman spectroscopy (LRS), high-magnification bronchoscopy (HMB), and high-definition bronchoscopy (HD) [43].

- NBI uses narrow-band filters that enhance the visualization of mucosal and submucosal vessels, and assess the abnormal angiogenesis seen in malignant lesions [44]. On NBI examination, tortuous and steeply terminated vessels are more common in squamous cell carcinoma [23].
- Optical coherence tomography (OCT) uses near infrared light, interacts with tissue architecture as a function of depth, and allows for cross sect ional imaging with specificity close to a histological examination by optical interferometry, with spatial resolution of 3 to 15 μm and a penetration depth of 2 mm m and a penetration depth of 2 mm [45]. This examination distinguishes squamous cell carcinoma, is complementary to AFB and NB distinguishes squamous cell carcinoma, is complementary to AFB and NBI [24].
- Confocal laser endomicroscopy and laser Raman spectroscopy (LRS) provide in vivo images below the bronchial surface and perform a detailed analysis of cells and images below the bronchial surface and perform a detailed analysis of cells and subcellular structures, highlighting neoplastic changes subcellular structures, highlighting neoplastic changes [24,46].
- Optical coherence tomography (OCT) uses near-infrared light, interacts with tissue architecture as a function of depth, and allows for cross-sectional imaging with specificity close to a histological examination by optical interferometry, with a spatial resolution of 3 to 15 (Table 4).

## 4. Discussion

In this review, we aimed to establish a correlation of clinical and paraclinical features for endobronchial squamous cell carcinoma. Clinical and paraclinical features were reviewed. These features are vaguely approached in lung cancer diagnosis and treatment guides.

Endobronchial squamous cell carcinoma is one of the most common tumors with a central location [26], being the most common type of cancer that is located inside the tracheobronchial tree [47]. Symptoms common to endobronchial tumors are cough, chest pain, recurrent pneumonia [3,4], atelectasis, post-obstructive pneumonia, wheezing [26], and hemoptysis, especially after bronchoscopy with biopsy [3,26], compared to other types of endobronchial tumors [3].

Usual laboratory analyses revealed the presence of hypercalcemia in the blood [26,28], compared to the assessment of analyses for lung adenocarcinoma. Analysis of blood tumor markers detected increased levels of SCC-Ag, CYFRA21-1, and CEA [6,15,16,17] in patients with squamous cell carcinoma of the lung, compared to those who presented with adenocarcinoma [15].

CT scans have highlighted the characteristic signs of endobronchial tumors, with different degrees of obstruction of the bronchial tree, the bronchus sign, and the cutoff sign, with the prominence of the endobronchial tumor formation [5,9,19,20,38,39,40]. On closer examination, there are signs of localized thickening of the bronchial wall in squamous cell carcinoma [19], but it is not mentioned in the literature as a specific feature.

Bronchoscopy with autofluorescence highlights the color changes at the level of the basement membrane in malignant formations, due to an abundant vascularization. Neoplastic lesions are represented as dark-red images due to mucosal hypertrophy that decrease fluorescence by increasing blood flow to the malignant tissue [25]. Squamous cell carcinoma in situ is detected by bronchoscopy with superficial autofluorescence, even if the basement membrane is not involved [47]. Most often, lesions can be detected in the central airways. Occasionally, the therapeutic approach may consist of bronchoscopic interventions [24]. Central squamous cell carcinoma lesions are known to invade the basement membrane in relation to the bronchus cartilage and do not invade it [24]. Squamous cell carcinomas confined to the bronchial wall show two distinct patterns of growth: superficial spread and endobronchial mass lesions [37]. Compared with lung squamous cell carcinoma, adenocarcinoma in situ, the preinvasive form, is mostly located in the lung parenchyma; it is usually diagnosed on resected tissue [24,25].

Bronchoscopy with narrow-band imaging (NBI) demonstrated tortuous blood vessels in 72% of patients with squamous cell carcinoma of the lung, compared with 8% in adenocarcinoma [23].

Taking biological biopsy samples of tumor tissue or suspected tumor tissue, by autofluorescence bronchoscopy, has a high accuracy in the diagnosis of anatomopathological certainty “gold standard” immunohistochemistry [48].

The confirmation of histologic diagnosis determines the surgical resection of early-stage disease, while pathologic grading and molecular testing allow for personalized tumor-type selection, adjuvant therapy, and a genotype-based treatment regimen, designed to improve survival in advanced-stage patients [13].

The recognition of clinical signs, circulating tumor markers, characteristics of computed-tomographic examination, and several types of bronchoscopic investigations are all involved in the early detection of this type of tumor. The approach of a personalized therapy, with a view to a surgical treatment to preserve the lung parenchyma, led us to carry out this review.

## 5. Limitations

The specialized literature has a small number of research articles on this topic.

## 6. Conclusions

Endobronchial squamous cell carcinoma is one of the most common tumors with endoluminal development. The assessment of clinical and paraclinical features can contribute to early detection. Patients can benefit from personalized therapy and lung-sparing surgery. The update of clinical and paraclinical characteristics of endobronchial squamous cell carcinoma opens new paths for scientific research with the aim of early detection. As a result, lung-sparing surgery, such as lung resection with broncho-anastomosis, will be practiced more frequently, leading to an increase in the quality of life of this type of patient.

## Figures and Tables

**Table 1:** Representative articles specifying the main author and the types of examination researched.

NR	Ref	Author	NR Patients	Study Type	Diagnostic Yeld	Sensitivity	Specificity	CT	Bronhoscopy/AFB	Bronhoscopy/NBI	Clinic	Tumor Merker
1	[3]	Saibin Wang	531	retrosp	95%	95%	95%	unspec	95%	unspec	CLINIC	unspec
2	[5]	Xiaochuan Zhang	366	retrosp	91.40%	91, 14%	92.40%	CT	unspec	unspec	CLINIC	unspec
3	[7]	Wei Zhao	135	retrosp	91.30%	unspec	unspec	unspec	unspec	unspec	unspec	MARKER
4	[8]	Suofu Ye	1922	retrosp	90.20%	82.50%	81.30%	unspec	unspec	unspec	unspec	MARKER
5	[9]	Tatsuya Imabayashi	1021	retrosp	75.9–95%	95%	95%	CT	AFB	unspec	unspec	unspec
6	[10]	Camelia Bădescu	156	prosp	84%	84%	84%	unspec	AFB	unspec	unspec	unspec
7	[11]	Zheng Liu	708	retrosp	89.30%	95.70%	89.30%	unspec	AFB	unspec	unspec	unspec
8	[12]	Urska Janzic	54	observ	72%	0	0	0	0	0	0	MARKER
9	[15]	Linjie Liu	2097	observ	65.93%	81.63%	65.93%	0	0	0	0	MARKER
10	[16]	Zhong-qing Chen	693	observ	65.93%	0	0	0	0	0	0	MARKER
11	[17]	Rafael Molina	3144	observ	0	88.50%	82.00%	0	0	0	0	MARKER
12	[18]	Viorel Biciușcă	38	descrip	0	0	0	0	AFB	0	CLINIC	0
13	[19]	Yoonah Song	5	retrosp	0	0	0	CT	AFB	0	0	0
14	[20]	Semra Bilaçeroğlu	92	prosp	68%	68%	68%	CT	AFB	0	0	0
15	[21]	Jiayuan Sun	232	meta-anal	95%	95%	95%	0	95%	95%	0	0
16	[22]	Xiaoxuan Zheng	218	retrosp	92.10%	92.10%	87.30%	0	AFB	0	0	0
17	[23]	Bojan Zaric	65	descrip	0	0	0	0	0	mucosal with tortuous blood vessels were identified in 72%	0	0
18	[24]	Ankit Gupta	0	review	95%	95%	95%	0	95%	mucosal with abnormal vascular patterns include increased vessel growth, tortuous vessels, dotted vessel, and spiral or screw-type vessels	0	0
19	[25]	Hongling Wang	38	descrip	93.4%	93.4%	93.4%	0	AFB	0	0	0

**Table 2:** The representative scientific studies that we analyzed for tumor markers alone or in groups, with significance for squamous cell carcinoma.

REFERENCES	[7]	[8]	[12]	[15]	[16]	[17]
**AUTHOR**	**Wei Zhao**	**Suofu Ye**	**Urska Janzic**	**Linjie Liu**	**Zhong-qing Chen**	**Rafael Molina**
**NR PATIENTS**	135	1922	54	2097	693	3144
**STUDY TYPE**	retrospective	retrospective	observational	observational	observational	observational
**DIAGNOSTIC YELD**	91.30%	90.20%	72%	65.93%	65.93%	unspecified
**SENSITIVITY**	unspecified	82.50%	unspecified	81.63%	unspecified	88.50%
**SPECIFICITY**	unspecified	81.30%	unspecified	65.93%	unspecified	82%
**SCC-Ag**	59.60%	unspecified	unspecified	36.68%	39.80%	4.80%
**CYFRA21-1**	unspecified	51%	unspecified	61.15%	88.60%	12.60%
**CEA**	55.80%	33.80%	unspecified	21.55%	23.50%	8.20%
**TrxR**	unspecified	71.60%	unspecified	unspecified	unspecified	unspecified
**NSE**	unspecified	21.30%	unspecified	7.51%	unspecified	17.20%
**bFGF**	65.40%	unspecified	unspecified	unspecified	unspecified	unspecified
**CEA + SCC-Ag + bFGF**	91.30%	unspecified	unspecified	unspecified	unspecified	unspecified
**CA19-9**	unspecified	18.80%	unspecified	unspecified	unspecified	unspecified
**NSE + Cyfra21-1 + CA19-9 + CEA + TrxR**	unspecified	83%	unspecified	unspecified	unspecified	unspecified
**NSE + Cyfra21-1 + CA 19-9**	unspecified	52.50%	unspecified	unspecified	5.54%	unspecified
**PD-L1**	unspecified	unspecified	72%	unspecified	unspecified	unspecified
**ProGRP**	unspecified	unspecified	unspecified	8.27%	unspecified	32.00%
**CA125**	unspecified	unspecified	unspecified	20.05%	28.90%	unspecified
**CEA + CYFRA21-1 + SCC-Ag + ProGRP + CA 125**	unspecified	unspecified	unspecified	65.93%	unspecified	unspecified
**CEA + CA125 + CA15-3 + CA19-9 + CA72-4 + CYFRA21-1 + SCC-Ag**	unspecified	unspecified	unspecified	unspecified	unspecified	unspecified
**CA72-4**	unspecified	unspecified	unspecified	unspecified	16.90%	unspecified
**CA 15-3**	unspecified	unspecified	unspecified	unspecified	9%	26.60%
**CA15.3 + CEA + CYFRA 21-1 + NSE + ProGRP**	unspecified	unspecified	unspecified	unspecified	unspecified	88.50%

**Table 3:** Representative scientific research analyzed for the specific elements of CT examinations.

NR	1	2	3	4
**REFERENCES**	[5]	[9]	[19]	[20]
**AUTHOR**	**Xiaochuan Zhang**	**Tatsuya Imabayashi**	**Yoonah Song**	**Semra Bilaçeroğlu**
**NR PATIENTS**	366	1021	5	92
**STUDY TYPE**	retrospect	retrospect	retrospect	prospect
**SENSITIVITY**	91.40%	95%	unspecif	68%
**SPECIFICITY**	92.40%	95%	unspecif	68%
**DIAGNOST YELD**	91.40%	95%	unspecif	68%
**POLIPOID LESSIONS**	present	present	unspecif	present
**FOCAL SESILE LESSIONS**	present	present	unspecif	present
**CT bronchus sign**	present	present	present	present
**Bronchial cutoff sign**	present	present	present	present
**PLATED LESSIONS OF THE WALL**	present	present	present	present

**Table 4:** The representative scientific research carried out by us for the bronchoscopy used in the discovery and biopsy of lung squamous cell carcinoma.

	1	2	3	4	5	6	7	8	9	10	11
**REFERENCES**	[3]	[9]	[10]	[11]	[18]	[20]	[21]	[22]	[23]	[24]	[25]
**AUTHOR**	**Saibin Wang**	**Tatsuya Imabayashi**	**Camelia Bădescu**	**Zheng Liu**	**Viorel Biciușcă**	**Semra Bilaçeroğlu**	**Jiayuan Sun**	**Xiaoxuan Zheng**	**Bojan Zaric**	**Ankit Gupta**	**Hongling Wang**
**NR PATIENTS**	531	1021	156	708	38	92	232	218	65	0	38
**STUDY TYPE**	retrosp	retrosp	prospect	retrospect	descriptive	prospective	meta-analysis	retrospect	descript	review	descript
**DIAGNOSTIC YELD**	95%	75.9%	84%	89.30%	0	68.00%	95.00%	92.10%	0	95%	93.4%
**SENSITIVITY**	95%	0	84%	95.70%	0	68%	95%	92.10%	0	95%	93.4%
**SPECIFICITY**	95%	0	84%	89.30%	0	68%	95%	87.30%	0	95%	93.4%
**BRONHOSCOPY/AFB**	0	0	84%	89.30%	0	0	95%	92.10%	0	95%	93.4%
**BRONHOSCOPY/NBI**	0	0	0	0	0	0	0	0	**NBI**	95%	0
**FIBEROPTIC BRONHOSCOPY**	exam	0	0	0	**exam**	**exam**	0	0	0	0	0
**WHITE LIGHT BRONHOSCOPY**	0	0	69.49%	75.60%	0	0	88.53%	62.20%	0	0	86.8%
**FLUOROSCOPIC BRONHOSCOPY**	0	**exam**	0	0	0	**exam**	0	0	0	0	0
**MUCOSAL CHANGES**	modified mucosa	modified mucosa	modified mucosa	99%	modified mucosa	modified mucosa	modified mucosa	modified mucosa	tortuous blood vessels were identified in 72%	abnormal vascular patterns include increased vessel growth, tortuous vessels, dotted vessel, and spiral or screw-type vessels	0

## Data Availability

Not applicable.
